# Dynamic Precipitation in Mg–8.08Gd–2.41Sm–0.30Zr Alloy during Hot Compression

**DOI:** 10.3390/ma11112147

**Published:** 2018-10-31

**Authors:** Limin Zhu, Quanan Li, Qing Zhang, Xiaoya Chen

**Affiliations:** 1 School of Materials Science and Engineering, Henan University of Science and Technology, 263, Kaiyuan Road, Luoyang 471023, China; zhulimin7705@163.com (L.Z.); foxzq@126.com (Q.Z.); 2 Collaborative Innovation Center of Nonferrous Metal, Henan Province, Luoyang 471023, China; 3 School of Materials Science and Engineering, Xi’an University of Technology, Xi’an 740048, China; chenxiaoya2010@163.com

**Keywords:** Mg–8.08Gd–2.41Sm–0.30Zr alloy, dynamic recrystallization, dynamic precipitation, hot compression

## Abstract

Dynamic precipitation of Mg–8.08Gd–2.41Sm–0.30Zr (wt %) alloy during hot compression was studied in the present work. The effects of temperature and strain rate on dynamic precipitation, and the effects of dynamic precipitation on dynamic recrystallization (DRX) and microhardness, were systematically analyzed. For this purpose, hot compression tests were conducted at the strain rates of 0.002~1 s^−1^ and temperatures of 350~500 °C, with the compaction strain of 70% (*ε*_max_ = 0.7). The obtained results revealed that dynamic precipitation occurred during hot compression at 350~400 °C, but did not occur for *T* ≥ 450 °C. The precipitates were demonstrated to be *β*-Mg_5_Gd with a size of 200~400 nm, and they were distributed in the DRXed region. Dynamic precipitation occurred at strain rates in the 0.002~0.01 s^−1^ range, but did not occur when the strain rates were in the 0.1~1 s^−1^ range for the hot compression temperature of 350 °C. The relationships between the hot compression temperature (*T*) and DRXed grain size (ln*d*), microhardness (*H_v_*), and DRXed grain size (*d*^−1/2^) of Mg–8.08Gd–2.41Sm–0.30Zr alloy were obtained.

## 1. Introduction

Mg–Gd alloys have been widely studied recently, due to their good mechanical properties and creep resistance [1,2,3]. These investigations have mainly focused on the strengthening method and its mechanism [4,5,6]. This research has recently been extended to the thermal deformation of Mg–Gd alloys [7,8,9,10]. It is known that the effective deformation modes of magnesium alloys are extrusion, forging, or rolling [11,12,13] and that the ductility and strength can be significantly enhanced due to the fine grains associated with dynamic recrystallization (DRX) [14,15,16,17], texture [10,18], and dynamic precipitation (DP) of rare earth compounds [14,19,20]. Small dispersed second phase particles are produced during dynamic precipitation and have significant effects on the DRX process and alloy properties. Therefore, some researchers have studied the dynamic precipitation of magnesium alloys during the deformation process. Chen [11] has studied the effect of rolling passes on the mechanical properties and microstructures of the Mg–Gd–Y–Zr alloy sheets. Dynamic precipitation was found in the hot rolling of Mg–Gd–Y–Zr alloy, and *β*’ phase precipitates were observed. The *β*’ phase increased with the increase in the rolling passes, while grain refinement and the (0002) basal texture weakened simultaneously. The *β* phase (Mg_5_Gd), found in Mg–1.8Gd–1.8Y–0.7Zn–0.2Zr (mol %) alloy by hot extrusion, was studied by Homma [18]. The *β* phase was obtained due to the aging and dynamic precipitation. However, the dynamic precipitation was not studied further. Xiao [19] has studied the dynamic precipitation behavior of Mg–Gd–Y–Zr alloys during hot deformation, and the results showed that the dynamic precipitation of *β*-Mg_5_(Gd,Y) was sensitive to the deformation temperature. Dynamic precipitation occurred in the temperature range of 300~400 °C during hot compression, but did not occur when the deformation temperature exceeded 450 °C. While the characteristics of the precipitates have been studied, the effects of dynamic precipitation on the properties have not been studied. Asqardoust [20] has studied the microstructural evolutions of a WE (W is Y element, and E is rare earth elements) magnesium alloy, and found that the dynamic precipitation of *β* phases leads to a flow softening at 350 °C, but the rare earth compounds, once again, dissolved into the matrix when the deformation temperature exceeded 450 °C. The present studies of dynamic precipitation have been carried out mainly for Mg–Gd–Y–Zr alloys, while the dynamic precipitation in Mg–Gd–Sm–Zr alloys has not been studied systematically to date. In particular, dynamic precipitation and its effects on the recrystallization process, and the relationship between the dynamic precipitation and the properties, has not been studied thoroughly.

In the present work, the temperature and strain rate for dynamic precipitation and its influence on DRX and properties were studied, and the model of dynamically recrystallized (DRXed) grain size-temperature, with dynamic precipitation, were constructed.

## 2. Experimental and Procedures

The chemical compositions of Mg–8.08Gd–2.41Sm–0.30Zr (wt %) alloy was analyzed using ICP-MS (inductively coupled plasma-mass spectrometry). Alloy ingots were prepared by melting the raw materials, namely pure Mg (99.9%) and the Mg–30Gd, Mg–30Sm, and Mg–30Zr (wt %) master alloys, in an induction melting furnace using an Al_2_O_3_ crucible in a mixed protection atmosphere of SF_6_ and CO_2_ with the volume ratio of 1:100. First, the magnesium ingot was melted at 700 °C, then, the Mg–30Gd, Mg–25Sm, and Mg–30Zr master alloys were added into the crucible. The molten metals were held for 5 min at 750 °C, and then poured into a metallic mold that was preheated to approximately 250 °C. The ingot was homogenized at 525 °C for 8 h, and then the specimens with a diameter and length of 10 and 15 mm, respectively, were machined.

A scanning electron microscope (SEM, JSM-5610LV, JEOL, Tokyo, Japan), equipped with an energy dispersive spectrometer (EDS, EDAX Inc., Mahwah, NJ, USA), was used to analyze the homogenized microstructures. Three areas were measured in the sample, and the three measurement results were averaged in the EDS test. Hot compression tests were performed at temperatures of 350~500 °C under strain rates of 0.002~1 s^−1^ using a thermomechanical simulator (Gleeble-1500, DSI, Poestenkill, NY, USA). Graphite was used as a high-temperature lubricant between the crossheads and the specimens. Prior to hot deformation, the specimens were heated to compression temperatures, and were held at that temperature for 3 min. After hot compression, the specimens were immediately quenched in water. The microstructural observations were performed on the longitudinal sections after etching in a solution of 100 mL of ethanol (99.7%) and 4 mL of nitric acid (65~68%). For the microstructural analysis of test alloys after the compression tests, an optical microscope (OM, Vert.A1, Carl Zeiss, Oberkochen, Jena, Thuringia, Germany) and SEM were used. To characterize the precipitates, a transmission electron microscope (TEM, JEM-2100, JEOL, Tokyo, Japan) was used. To study the relationship between the size of the recrystallized grains and the dynamic precipitation, electron backscatter diffraction (EBSD) observation was conducted using a JSM-7800 SEM (JEOL, Tokyo, Japan) equipped with an HKL-EBSD system. The grain size of DRX was analyzed using Channel 5 software (HKL, Oxford, Oxfordshire, UK). More than 150 grains were used for statistical grain size determination. The Vickers hardness (*H_v_*) tests of the DRXed zone were performed with a microhardness apparatus (MH-3, Heng Yi, Shanghai, China) using a 100 g load for 10 s. More than 10 datapoints were measured for each sample, for statistics.

## 3. Results

### 3.1. Initial Microstructure

**Figure 1:**
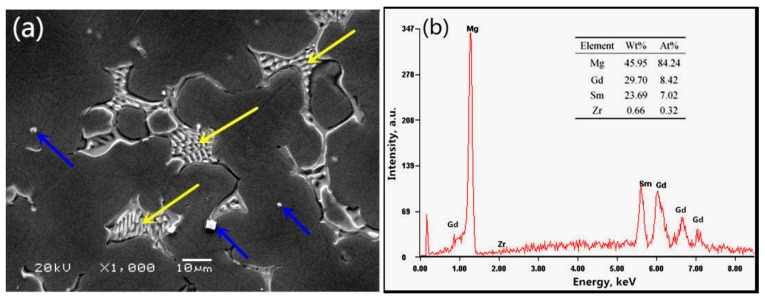
The SEM (**a**) and EDS (**b**) results for the Mg–8.08Gd–2.41Sm–0.30Zr as-cast alloy.

**Figure 2:**
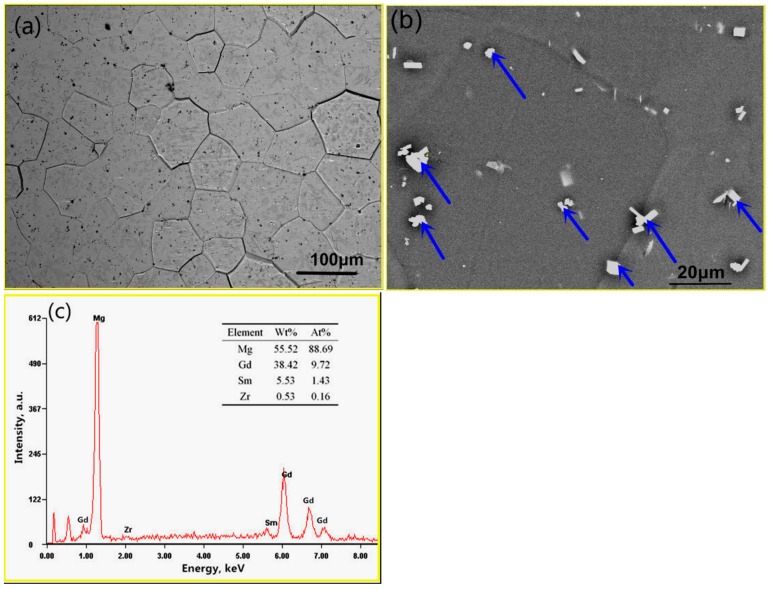
The optical micrograph of homogenized Mg–8.08Gd–2.41Sm–0.30Zr alloy (**a**), BSE image (**b**), and EDS (**c**).

The SEM and EDS results for the Mg–8.08Gd–2.41Sm–0.30Zr as-cast alloy are shown in Figure 1. Coarse eutectic structures, rich in Gd and Sm rare earth elements, are observed (marked by yellow arrows in Figure 1a). There are some block-shaped phases inside the grains (marked by blue arrows in Figure 1a). After solution treatment, the microstructure of the alloy consists of equiaxed grains with the average size of ~80 μm (measured using the quantitative metallography method), and the block-shaped phases dispersed in the matrix (as shown in Figure 2a). Most of the eutectic phases dissolved into the magnesium matrix during the homogenization treatment. However, there are still some undissolved micron-size block-shaped phases in the matrix (marked by blue arrows in Figure 2b). EDS analysis shows that these block-shaped phases are rich in Gd and Sm rare earth elements (Figure 2c). They may be the high melting point compounds produced during casting.

### 3.2. Dynamic Precipitation

**Figure 3:**
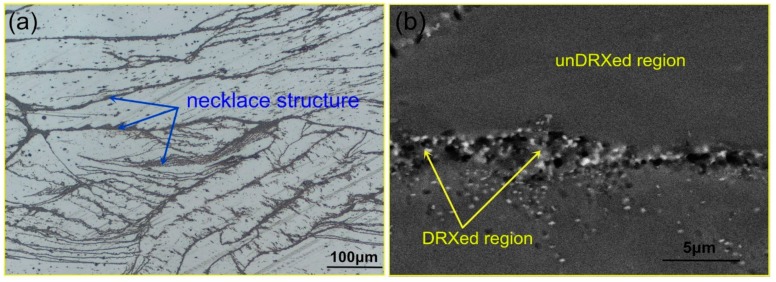
The optical micrograph (**a**) and BSE image (**b**) of the Mg–8.08Gd–2.41Sm–0.30Zr alloy deformed under the conditions of $\dot{\varepsilon }$ = 0.002 s^−1^ and *T* = 350 °C.

**Figure 4:**
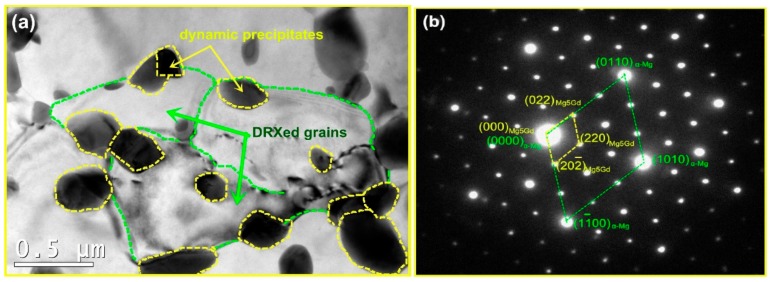
The TEM image of DRXed region (**a**) and the selected area electron diffraction (SAED) pattern (**b**).

Figure 3a shows the optical micrograph of Mg–8.08Gd–2.41Sm–0.30Zr alloy deformed under the conditions of $\dot{\varepsilon }$ = 0.002 s^−1^ ($\dot{\varepsilon }$ strain rate) and *T* = 350 °C. The original grains of the homogenized alloy are stretched perpendicular to the compression direction. Necklace structures are observed along the deformed grain boundary. The necklace structures are considered to be fine DRXed grains, as is demonstrated in Figure 4a. Figure 3b shows the BSE image of the same specimen. Numerous nanoparticles are found in the DRXed regions (deformed grain boundary), but not in the unDRXed regions. This shows the dynamic precipitation in the process of hot deformation, which is generated during DRX. A large number of holes are also observed in the DRXed regions that may be caused by the migration and precipitation of solute atoms, or by the dissolution of impurities in the grain boundary during the corrosion process.

**Figure 5:**
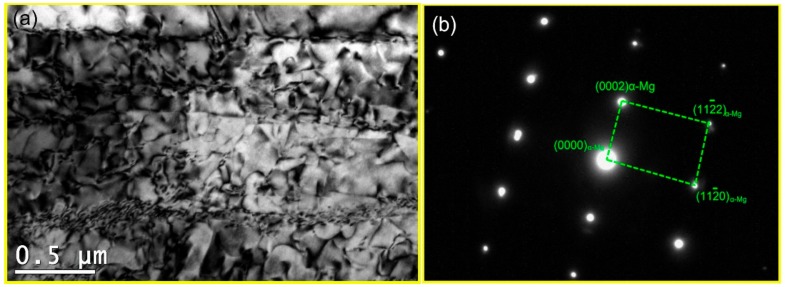
The TEM image of unDRXed region (**a**) and the SAED pattern (**b**).

**Figure 6:**
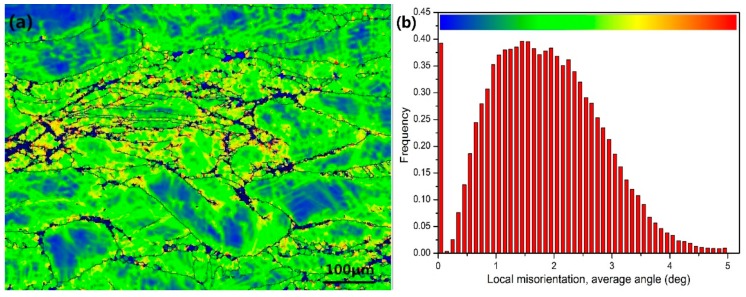
The local misorientation map (**a**) and (**b**) the frequency of the local misorientation (350 °C, 0.01 s^−1^).

The TEM micrograph of the DRXed region and the selected area electron diffraction (SAED) pattern are shown in Figure 4. It is observed that dynamic precipitation occurs with DRX during the compression. The precipitated second phase particles, with sizes in the 200~400 nm range, are distributed in the grain boundary of the DRX, giving rise to the DRXed grain refinement. The grain size of the DRX is in the approximate range of 1~2 μm. The precipitate is *β*-Mg_5_Gd with a face-centered cubic structure. Dynamic precipitation does not occur in the unDRXed regions, as shown in Figure 5. Particles are not observed in this region but, rather, some dislocation tangles and a large number of basal slip lines are found. Sasaki [14] stated that the preferred nucleation sites for dynamic precipitation are provided by dislocation tangles. However, in this study, the dynamic precipitation is observed at the grain boundaries, but not in the dislocation-enriched regions. This is because the strain at the grain boundary is greater than that in the intragranular region. Compounds are prone to nucleation because of the higher deformation storage energy. Figure 6a shows the local misorientation map (350 °C, 0.01 s^−1^). The frequency of the local misorientation and rainbow color are shown in Figure 6b. The misorientation is larger in the grain boundary of the deformation region (red regions in Figure 6a), and misorientation is lower in the intragranular regions (blue regions in Figure 6a). The strain is proportional to the misorientation. This means that the strain and the stored deformation energy are greater at the grain boundaries. Nucleation of both dynamic precipitation and DRX is easier at the grain boundaries. This is consistent with Saboori’s findings [21]. Saboori demonstrated that the new DRX grains nucleate and grow at the deformed and strained high-angle interfaces.

## 4. Discussion

### 4.1. Effect of Temperature on Dynamic Precipitation

**Figure 7:**
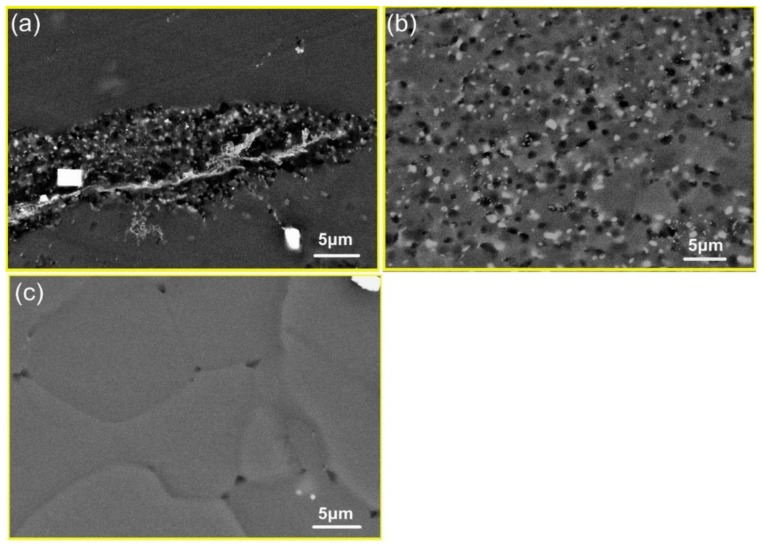
The BSE images of specimens deformed at (**a**) $\dot{\varepsilon }$ = 0.002 s^−1^ and *T* = 350 °C, (**b**) $\dot{\varepsilon }$ = 0.002 s^−1^ and *T* = 400 °C, and (**c**) $\dot{\varepsilon }$ = 0.002 s^−1^ and *T* = 450 °C.

**Figure 8:**
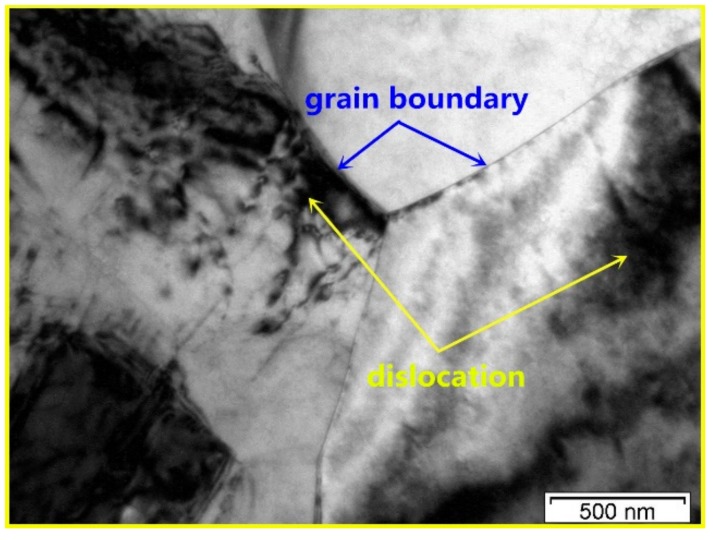
The TEM image of specimen deformed at $\dot{\varepsilon }$ = 0.002 s^−1^ and *T* = 450 °C.

**Figure 9:**
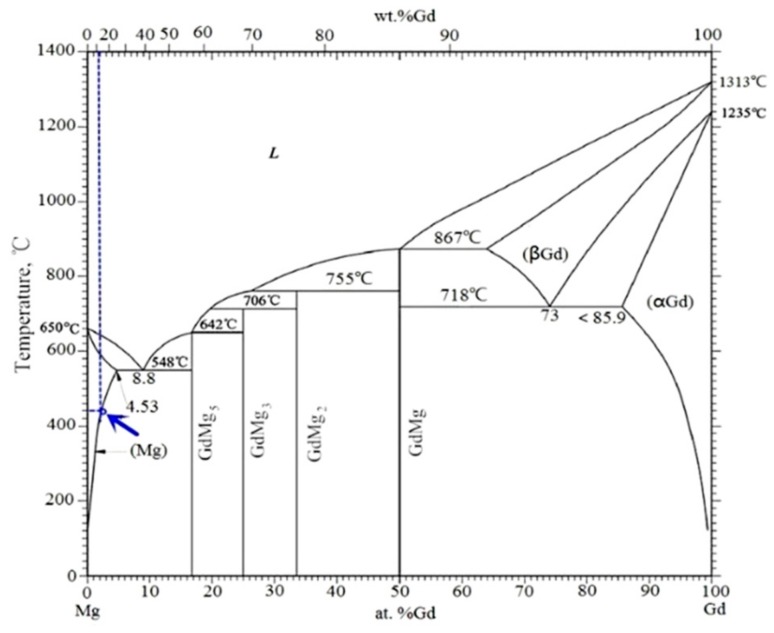
The Mg–Gd phase diagram.

Figure 7 shows the BSE images of Mg–8.08Gd–2.41Sm–0.30Zr alloy after hot compression at the strain rate of 0.002 s^−1^ and different temperatures. The nanosized particles precipitated in the DRXed region when the hot temperature was 350 °C (Figure 7a). As the deformation temperature increased, the dynamic precipitated particles grew (Figure 7b). When the hot temperature increased to 450 °C, no precipitated particles were found, and only larger DRXed grains were observed (Figure 7c). The TEM image of the specimen deformed at 0.002 s^−1^ and 450 °C is shown in Figure 8. It is observed that the DRXed grain boundary is smooth, and many dislocations are present in the grains. As observed from the above experimental results, the deformation temperature has a significant influence on dynamic precipitation. Dynamic precipitation occurred in the temperature range of 300~400 °C during hot compression, but did not occur when the deformation temperature exceeded 450 °C. This process can be illustrated using the Mg–Gd and Mg–Sm phase diagram [22]. Figure 9 shows the Mg–Gd phase diagram. With the increase in the temperature, the solubility of Gd in *α*-Mg increases gradually. When the Gd content is 10~11 wt % (Sm and Gd contents were estimated for all cases), the solubility curve corresponds to the temperatures in the 420~450 °C range (marked by the blue arrow in Figure 9). This means that the Mg_5_Gd phase is dissolved when the temperature is higher than this temperature. However, the phase transition during hot compression is a non-equilibrium phase transition, and the precipitation and dissolution processes are different from the equilibrium phase diagrams. Therefore, it is necessary to study the dynamic precipitation during the thermal deformation in detail.

### 4.2. Effect of Strain Rate on Dynamic Precipitation

**Figure 10:**
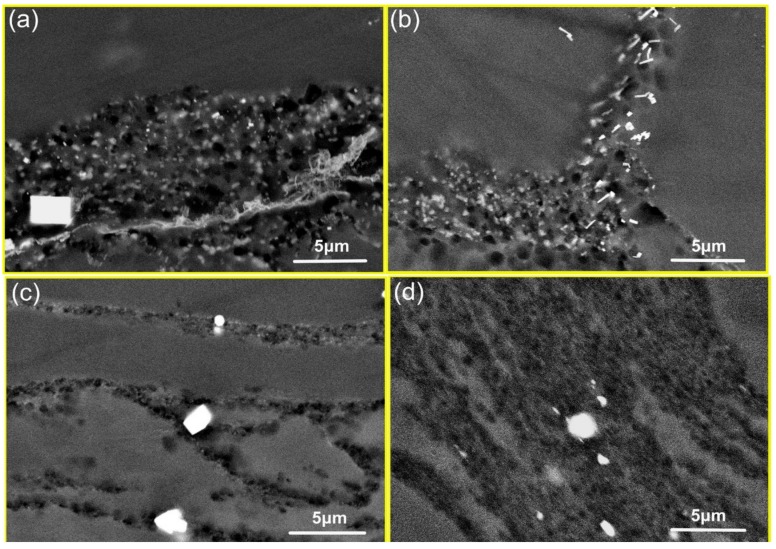
The BSE images of specimens deformed at (**a**) $\dot{\varepsilon }$ = 0.002 s^−1^ and *T* = 350 °C, (**b**) $\dot{\varepsilon }$ = 0.01 s^−1^ and *T* = 350 °C, (**c**) $\dot{\varepsilon }$ = 0.1 s^−1^ and *T* = 350 °C, and (**d**) $\dot{\varepsilon }$ = 1 s^−1^ and *T* = 350 °C.

Figure 10 shows the BSE images of Mg–8.08Gd–2.41Sm–0.30Zr alloy after hot compression at 350 °C and the different strain rates. Dynamic precipitation is observed at the strain rate of 0.002~0.01 s^−1^ (Figure 10a,b). A large number of fine secondary phase particles are observed in the grain boundary regions of the deformed grains (DRXed regions). As the strain rate increased to 0.1~1 s^−1^, no precipitates were observed in the BSE images, as shown in Figure 10c,d. This indicates that not only the temperature but, also, the strain rate influences the precipitation process.

The precipitation process includes the stages of nucleation and growth. The nucleation requires a certain temperature and a suitable nucleation site and, then, a certain time is required for the growth of the nucleus. For the experimental alloy, the dynamic precipitation temperature is 350~400 °C. The nucleation occurs preferentially at the grain boundaries, rather than the dislocation entanglement area, as confirmed by an examination of Figure 4 and Figure 5. For the strain rates of 1, 0.1, 0.01, and 0.002 s^−1^ (*T* = 350 °C and *ε* = 0.7), the deformation times are 0.7, 7, 70, and 360 s, respectively. Apparently, due to the short time, dynamic precipitation does not occur for the strain rates of 1 and 0.1 s^−1^.

### 4.3. Effect of Dynamic Precipitation on DRX

**Figure 11:**
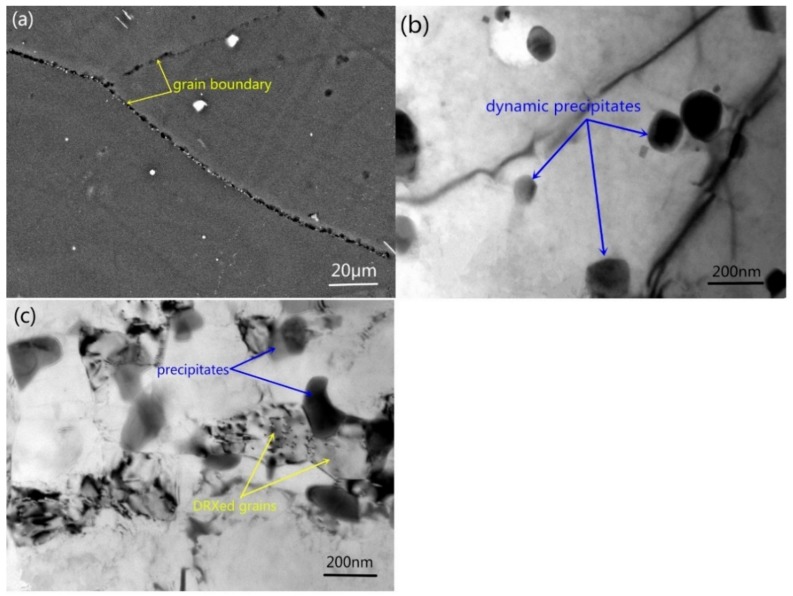
(**a**) BSE and (**b**) TEM images of the samples deformed to *ε* = 0.1, (**c**) TEM images of the sample deformed to *ε* = 0.3 (350 °C, 0.01 s^−1^).

To study the relationship between dynamic precipitation and DRX, the samples deformed at 350 °C and 0.01 s^−1^ to strain levels of 0.1 and 0.3 were analyzed (the critical strain for the occurrence of DRX was 0.246, as determined based on the true stress and true strain). The BSE and TEM images of the samples deformed to *ε* = 0.1 are shown in Figure 11a,b. Some fine precipitates are observed in the grain boundaries (Figure 11a), and no DRXed grains are found (Figure 11b). However, DRXed grains are observed in the sample deformed to 0.3 (Figure 11c). This indicates that the dynamic precipitation occurs prior to DRX.

The size of the DRXed grains is determined by the strain rate and deformation temperature. With the increase in the deformation temperature, the grain boundary migrates rapidly, and the grain grows easily. With increasing strain rate, the deformation energy can accumulate rapidly, promoting the acceleration of recrystallization nucleation and the refinement of DRXed grains. In addition to the influence of the thermal process on the grain size, the composition and structure of the alloy also have a significant influence on the size of the DRXed grains. The relationship between dynamic precipitation and the DRXed grain size in hot compression of magnesium alloy was analyzed.

The *Zener–Hollomon* parameter (*Z*), that is widely used to analyze the grain size of a hot deformation alloy [23,24], can be related to the strain rate and temperature, as shown in Equation (1).

(1) $$Z=\dot{\varepsilon }\exp(\frac{Q}{RT})\;$$

where $\dot{\varepsilon }$ is the strain rate, *Q* is the deformation activation energy, *R* is the universal gas constant, and *T* is the absolute temperature.

**Figure 12:**
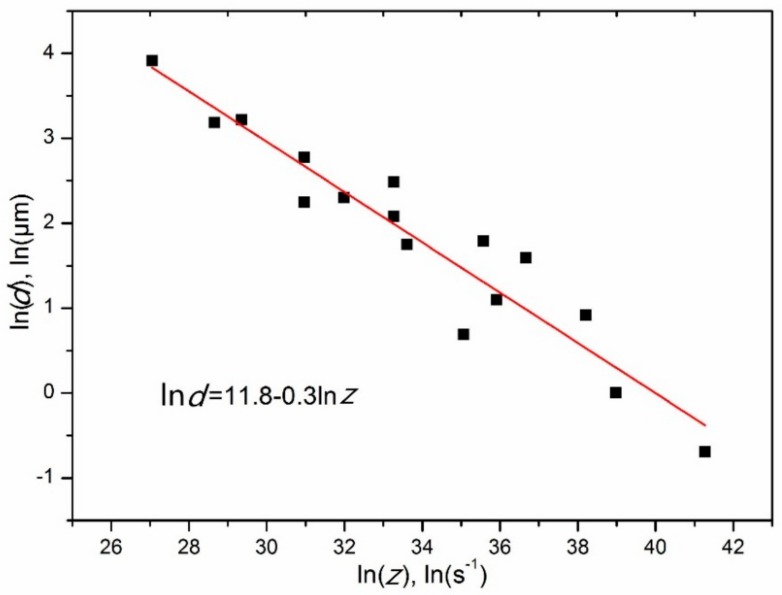
Plots for the relationship between the resulting grain size and *Zener*–*Holloman* parameter.

The relationship between the average DRXed grain size (*d*, in μm) and the *Zener–Hollomon* parameter for Mg–8.08Gd–2.41Sm–0.30Zr alloy was established, as shown in Figure 12, and is given quantitatively by

(2) lnd=11.8−0.3lnZ.

The relationship between the grain size and the *Zener*–*Hollomon* parameter is consistent with Chang’s findings [25], as shown in Equation (3).

(3) lnd=b−klnZ,

where *b* and *k* are constants (*b* > 0, *k* > 0). By substituting Equation (1) into Equation (3), the following relationship can be derived:

(4) $$\ln d=b-k\ln\dot{\varepsilon }-kQ/RT.$$

**Figure 13:**
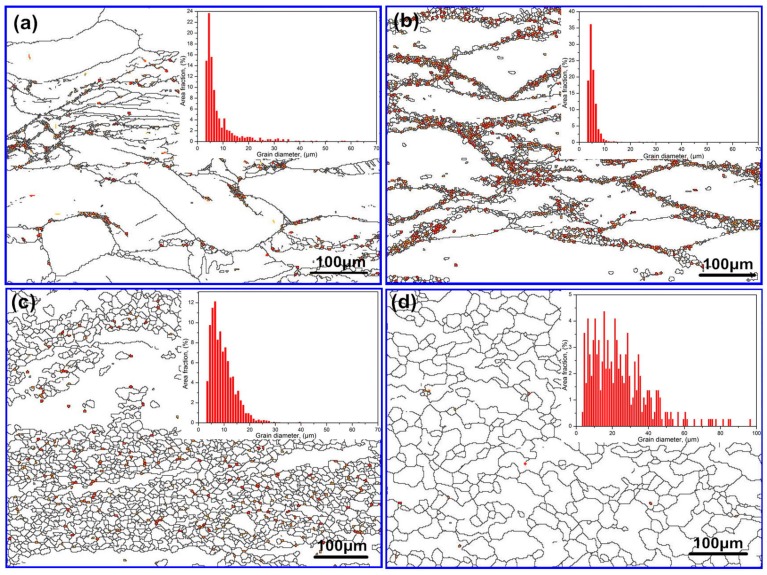
The boundary misorientation maps of the Mg–8.08Gd–2.41Sm–0.30Zr ($\dot{\varepsilon }$ = 0.01 s^−1^) (**a**) 350 °C, (**b**) 400 °C, (**c**) 450 °C, and (**d**) 500 °C.

**Figure 14:**
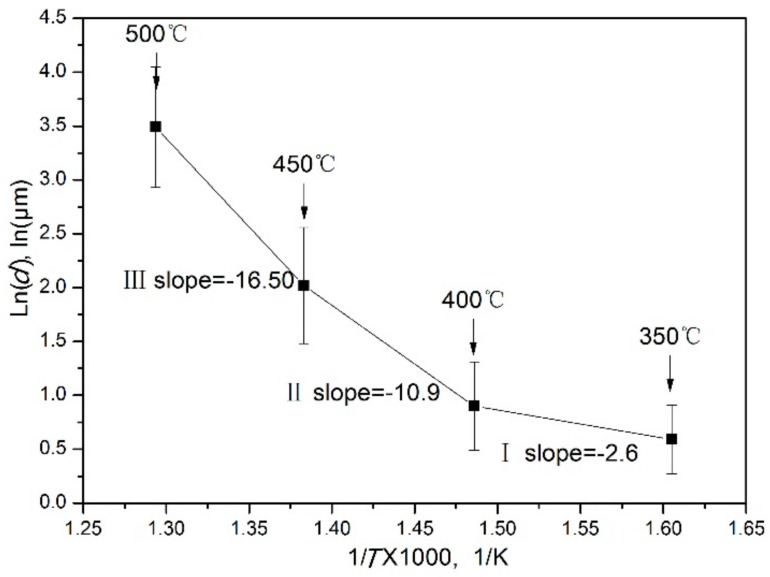
The relationship between ln*d* and 1/*T* at strain rate of 0.01 s^−1^.

The boundary misorientation maps of Mg–8.08Gd–2.41Sm–0.30Zr alloys, deformed at 0.01 s^−1^ and different temperature, are shown in Figure 13. Elongated grains and a small amount of the DRXed grains (necklace structures) are observed in Figure 13a,b. The frequency and grain size of the DRXed grains increase with the increasing temperature, as shown in Figure 13c,d. DRXed grains are measured using Channel 5 software. The relationship between ln*d* and 1/*T* is shown in Figure 14.

Surprisingly, the ln*d*–1/*T* curves measured at 0.01 s^−1^ do not show any linear dependence. This means that the grains grow at different speeds in different temperature ranges. The plot can be divided into three regions, as shown in Figure 14. This is due to the effects of dynamic precipitation on the size of the DRXed grains. The test temperature promoted grain growth, while the small second phase of dynamic precipitation hindered grain growth. Region I represents the temperature range of 350~400 °C. In this region, the slope is −2.6. The DRXed grains are fine. The average grain size increases slowly with the increasing temperature. The second phase particles of dynamic precipitation hinder the DRX grain boundary movement, due to the pinning effect, so that smaller DRXed grains are obtained [26,27,28]. This can be interpreted by TEM micrographs (Figure 4). Hence, in region I, the grain size of DRX is mainly controlled by the second phase particles. When the temperature increases from 400 °C to 450 °C, i.e., in region II, the average grain size increased markedly with increasing temperature. The slope is −10.9. In this region, the dynamic precipitation is weakened, and the particle size of the precipitate increases. The pinning effect on the grain boundary decreases, and the temperature effect is enhanced. Therefore, grain growth is easier in region II than in region I, as can be explained by Figure 7b,c. In region III, no dynamic precipitation occurred in the temperature range of 450~500 °C. The grain size is controlled by the temperature. Therefore, the DRXed grain size increases sharply in region III, due to the higher temperature and the absence of the pinning effect of the second phase particles.

**Figure 15:**
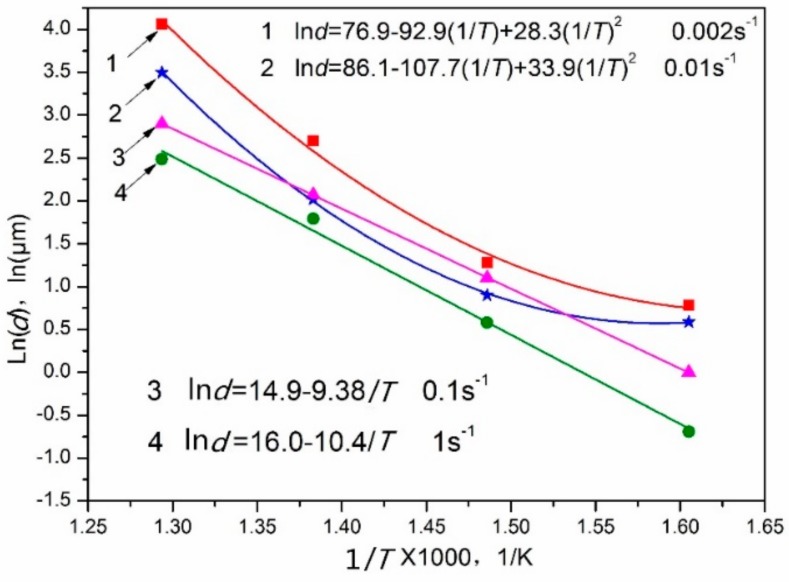
The relationships between ln*d* and 1/*T* at different strain rates.

The relationships between ln*d* and 1/*T* at different strain rates are shown in Figure 15. A nonlinear relationship is observed between ln*d* and 1/*T* at the 0.002~0.01 s^−1^ range, due to dynamic precipitation. Dynamic precipitation does not occur at the strain rates in the 0.01~1 s^−1^ range (Figure 10c,d); hence, a linear relation is observed between ln*d* and 1/*T* in this strain rate range.

### 4.4. Effect of Dynamic Precipitation on the Hardness

**Figure 16:**
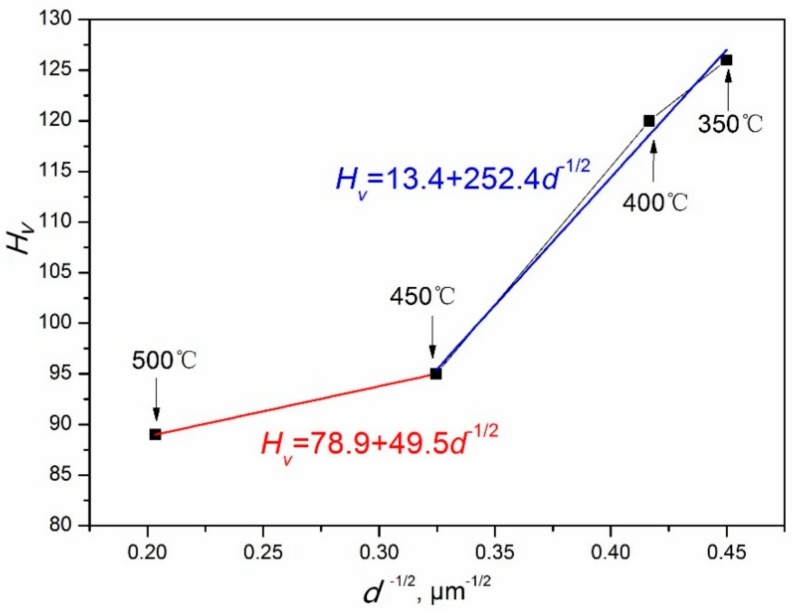
The relationship between *d*^−1*/*2^ and *H_v_*.

Chang [25] studied the relationship of the DRXed grain size and microhardness of AZ31 magnesium alloy. The DRX of AZ31 magnesium alloy is not affected by dynamic precipitation; hence, it is consistent with the *Hall*–*Petch* relationship given by *H_v_* = 40 + 72*d*^−1/2^. This can be observed from the above research results for the DRX and dynamic precipitation, occurring simultaneously in the hot compression process of Mg–8.08Gd–2.41Sm–0.30Zr alloys. Hence, the relationship between the hardness and grain size of DRX may not be completely in accordance with the *Hall*–*Petch* relationship. The microhardness of the DRXed regions were measured, and the relationship between the DRXed grain size and the microhardness was plotted, as shown in Figure 16. The studied specimens underwent hot compression at the strain rate of 0.01 s^−1^, and temperatures of 350~500 °C. For the temperatures of 450~500 °C and 350~450 °C, the *Hall*–*Petch* relationship (*H_v_* = *a* + *kd*^−1*/*2^) follows *H_v_* = 78.9 + 49.5*d*^−1*/*2^ and *H_v_* = 13.4 + 252.4*d*^−1*/*2^, respectively. When the temperature was between 350~400 °C, the average DRXed grain size was smaller, because of the pinning effect of the dynamic precipitated phase, and the microhardness values were higher (120~126 *H_v_*). With the temperature increasing (*T* > 400 °C), the dynamic precipitated phase gradually dissolved, and the microhardness value decreased. In the temperature range of 450~500 °C, the microhardness values are 89~95 *H_v_*, which are significantly lower than that of 350~400 °C. This is due to DRXed grain growing, and without any effect of dynamic precipitated phase. Due to the influence of dynamic precipitation, the microhardness and DRXed grain size of Mg–8.08Gd–2.41Sm–0.30Zr alloy are not consistent with the *Hall*–*Petch* relationships in the 350~500 °C temperature range.

## 5. Conclusions

The dynamic precipitation of Mg–8.08Gd–2.41Sm–0.30Zr alloy during hot compression was investigated. The effects of the temperature and strain rate on dynamic precipitation were analyzed. Additionally, the effects of dynamic precipitation on DRX and microhardness were studied. The following conclusions can be drawn:

(1) Dynamic precipitation occurs during hot compression at 350~400 °C, but does not occur at *T* ≥ 450 °C. The precipitates are demonstrated to be *β*-Mg_5_Gd with the size of 200~400 nm, and they are distributed in the DRXed region.
(2) Dynamic precipitation occurs at strain rates in the 0.002~0.01 s^−1^ range, but does not occur in the 0.1~1 s^−1^ range for the hot compression temperature of 350 °C. A nonlinear relationship is observed between DRXed grain size (ln*d*) and the hot compression temperature (1/*T*) at the 0.002~0.01 s^−1^ range, due to dynamic precipitation.
(3) The microhardness values of Mg–8.08Gd–2.41Sm–0.30Zr alloy are higher at temperatures of 350~400 °C, due to the influence of dynamic precipitation. With the temperature increasing (*T* > 400 °C), the dynamic precipitated phase gradually dissolves, and the microhardness value decreases.
